# *C. elegans* Nucleostemin Is Required for Larval Growth and Germline Stem Cell Division

**DOI:** 10.1371/journal.pgen.1000181

**Published:** 2008-08-22

**Authors:** Michelle M. Kudron, Valerie Reinke

**Affiliations:** Department of Genetics, Yale University School of Medicine, New Haven, Connecticut, United States of America; Stanford University Medical Center, United States of America

## Abstract

The nucleolus has shown to be integral for many processes related to cell growth and proliferation. Stem cells in particular are likely to depend upon nucleolus-based processes to remain in a proliferative state. A highly conserved nucleolar factor named nucleostemin is proposed to be a critical link between nucleolar function and stem-cell–specific processes. Currently, it is unclear whether nucleostemin modulates proliferation by affecting ribosome biogenesis or by another nucleolus-based activity that is specific to stem cells and/or highly proliferating cells. Here, we investigate nucleostemin (*nst-1*) in the nematode *C. elegans*, which enables us to examine *nst-1* function during both proliferation and differentiation in vivo. Like mammalian nucleostemin, the NST-1 protein is localized to the nucleolus and the nucleoplasm; however, its expression is found in both differentiated and proliferating cells. Global loss of *C. elegans* nucleostemin (*nst-1*) leads to a larval arrest phenotype due to a growth defect in the soma, while loss of *nst-1* specifically in the germ line causes germline stem cells to undergo a cell cycle arrest. *nst-1* mutants exhibit reduced levels of rRNAs, suggesting defects in ribosome biogenesis. However, NST-1 is generally not present in regions of the nucleolus where rRNA transcription and processing occurs, so this reduction is likely secondary to a different defect in ribosome biogenesis. Transgenic studies indicate that NST-1 requires its N-terminal domain for stable expression and both its G1 GTPase and intermediate domains for proper germ line function. Our data support a role for *C. elegans* nucleostemin in cell growth and proliferation by promoting ribosome biogenesis.

## Author Summary

Stem cells are carefully poised between the alternate fates of proliferation and differentiation. The regulation of this choice is a complex one that occurs on many different levels. One major influence controlling this choice derives signals emanating from the nucleolus, which serves dual roles as the site of ribosome biogenesis and as a repository for sequestered key regulatory factors. The nucleolar GTPase nucleostemin has recently been identified as a potential link between stem cell proliferation and nucleolar function, but its exact role in the nucleolus has not been directly addressed in a metazoan. Here, we use the model organism *C. elegans* to investigate the function of nucleostemin in both differentiated cells and proliferating stem cells. We show that nucleostemin probably acts to regulate ribosome biogenesis, and through this process controls cell proliferation. We also suggest that, at least in *C. elegans*, the function of nucleostemin is not restricted to proliferating stem cells, but that it also functions in differentiated cells to control cell growth. Our study highlights the complexity of the role of the nucleolus in regulation of cell growth and division.

## Introduction

The nucleolus is a dynamic structure to which an increasing diversity of functions is ascribed. Previously known primarily as the central site of ribosome subunit biosynthesis, the nucleolus has recently been recognized as a coordination center for many processes related to cell growth and proliferation, in addition to ribosome biogenesis [1]. The nucleolus changes size and appearance in response to metabolic and growth cues received by the cell when in interphase, suggesting that active signaling between external stimuli and the nucleolus exists. It can also serve as a repository for proteins with a wide variety of functions related to cell proliferation and genome integrity. For instance, key cell cycle proteins such as CDC14 are regulated by their coordinated release from the nucleolus at certain stages of the cell cycle [2].

Stem cells in particular are likely to rely on nucleolar regulation of cellular growth and proliferation. Regulation of telomerase activity is critical for the ability of stem cells to undergo self-renewing divisions and human telomerase reverse transcriptase (hTERT) can be found in the nucleolus at certain stages of the cell cycle [3]. Additionally, nucleolar mechanisms that permit rapid and robust responses to genotoxic stress are also likely to be especially prominent in stem cells, where maintaining integrity of the genome is of paramount importance [4]. For example, in response to DNA damage, RNA polymerase I activity in the nucleolus is down-regulated [5], and proteins that inhibit p53 are sequestered in the nucleolus [6]. Stem cells are delicately balanced between division and differentiation, and the nucleolus is a convenient place to hold transcription factors that affect differentiation. For instance, the transcription factor Hand1 is sequestered in the nucleolus of trophoblast stem cells, and its release directs those cells to differentiate into giant cells [7]. Finally, Polycomb factors are sequestered in the nucleolus during *Drosophila* spermatogenesis to permit differentiation of primary spermatocytes into mature spermatids [8].

A recently identified, highly conserved factor named nucleostemin is a potential link between nucleolar function and stem cell-specific processes [9]. It is preferentially expressed in stem cells and other proliferating cells, and shuttles between the nucleolus and nucleoplasm via its GTPase activity in a cell cycle-dependent manner [10]. Depletion or overexpression of nucleostemin in cell culture impairs normal cell proliferation [9]. Whether nucleostemin modulates some aspect of ribosome biogenesis or whether it has a different function that is more specific to stem cells and other rapidly proliferating cell types, remains unresolved. Mammalian nucleostemin is not present in the portion of the nucleolus where ribosomal RNA synthesis and processing occur [11], suggesting that it does not have a role in initial aspects of ribosome biogenesis. However, the class of nucleolar GTPases to which nucleostemin belongs includes yeast Nug1, which acts to export pre-60S ribosomal subunits out of the nucleolus [12]. Additionally, it is not known in which compartment of the cell nucleostemin function is actually required, or whether it is the act of shuttling between the nucleolus and nucleoplasm itself that is the critical activity. If the latter, cargo or associated proteins have remained unidentified. Thus, how nucleostemin might function in the nucleolus in proliferating cells is unknown.

We have investigated nucleostemin function in somatic and germline stem cell division in the nematode *C. elegans*, a multicellular organism that permits the study of nucleostemin during both proliferation and differentiation in vivo. Previous gene expression profiling experiments in our lab indicated that *C. elegans* nucleostemin, here named *nst-1*, is preferentially expressed in proliferating germ cells (unpublished data). We found that global loss of nucleostemin (*nst-1*) resulted in a larval arrest phenotype and failure of growth. Loss of *nst-1* specifically in the germ line, but not the soma, caused germline stem cells to fail to proliferate. Ribosomal RNA production is decreased in *nst-1* mutants prior to any obvious defects in the animal, suggesting that *nst-1* is required for ribosome biogenesis. NST-1 protein is localized to the nucleolus and the nucleoplasm, but in contrast to mammalian nucleostemin, NST-1 is found in both terminally differentiated, non-cycling cells as well as in proliferating cells. Transgenic studies indicated that NST-1 requires its N-terminal domain for stable expression, and requires both the G1 GTPase and intermediate domains for function in the germ line. Our results suggest that nucleostemin regulates ribosome biogenesis to mediate its effects on cell growth and proliferation.

## Results

### Lack of *nst-1* Causes Larval Arrest due to a Growth Defect

**Figure 1:**
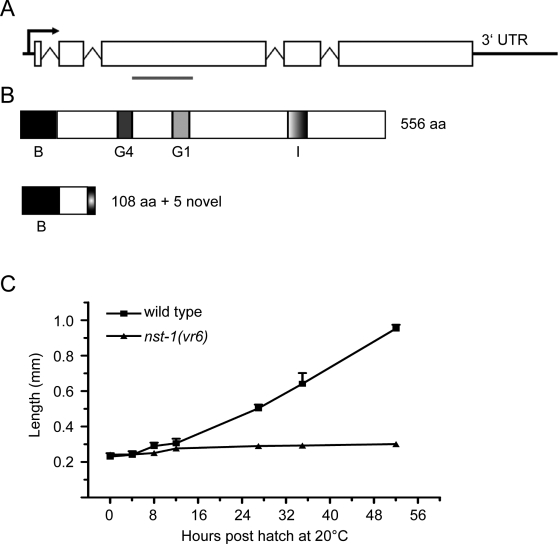
*nst-1* locus and mutant phenotype. A. Schematic of the *nst-1(vr6)* deletion. Region of deletion marked with gray line, stop codon occurs five amino acids after the end of the deletion. B. Diagram of the NST-1 protein and the predicted functional domains. B = basic domain, G4 = G4 GTPase domain, G1 = G1 GTPase domain, I = intermediate domain. Below is the predicted protein product resulting from the *nst-1(vr6)* deletion. C. *nst-1(vr6)* mutants were compared to wild-type animals by measuring their length from head to tail. Error bars, standard deviation (n = 6 per genotype).

To functionally characterize *nst-1*, we isolated an *nst-1* deletion mutant, *vr6*, by PCR screening of a mutagenized worm library. The *vr6* deletion allele is predicted to produce a truncated protein containing 108 N-terminal amino acids, followed by five novel amino acids before introduction of a stop codon, thus removing both GTPase domains (Figure 1A, B). RT-PCR analysis of *nst-1(vr6)* mutants detected only the truncated transcript, at levels that were four-fold reduced compared to wild type (Figure S1). *nst-1(vr6)* homozygous mutants born from heterozygous mothers arrest as young larvae in the L1 or L2 stage of development. The arrested larvae can live over 20 days, which is comparable to wild-type lifespans. The larvae are active and appear to feed normally.

Because all *nst-1(vr6)* homozygotes are born from heterozygous mothers, any requirement for *nst-1* activity in the embryo could be masked by maternally-deposited *nst-1* product. In order to determine if *nst-1* acts in embryos, we injected adult wild type and *nst-1* heterozygous animals with *nst-1* dsRNA to deplete both maternal and zygotic *nst-1* gene product, and assessed the progeny for possible phenotypes. No embryonic lethality was noted. Instead, the progeny uniformly arrested as L1 or L2 larvae, phenocopying *nst-1(vr6)* homozygotes (Table 1). These results suggest that wild type levels of maternal (or zygotic) *nst-1* are not essential for embryonic development, but are necessary for larval development. Moreover, this observation confirms that the deletion mutant phenotype is most likely due to the *nst-1* lesion and not an independent background mutation. Based on the RT-PCR data and the fact that *nst-1(vr6)*, *nst-1(RNAi)*, and *nst-1(RNAi); nst-1(vr6)/*mIn1 animals all display similar phenotypes, we suggest that the *nst-1(vr6)* deletion represents a strong loss-of-function or null mutation.

**Table 1:** Percent sterility and larval arrest of strains used.

Genotype	% Sterility	% Larval arrest	n
Wild type (N2)	0	0	>200
*nst-1(vr6)/*mIn1	0	0	>1000
*nst-1(vr6)*	0	100	>2800
*nst-1(RNAi)*; wild type (N2)	0	100	>1141
*nst-1(RNAi); nst-1(vr6)/*mIn1	0	100	>150
*rrf-1(pk1417)*	0	0	>80
*rrf-1(pk1417); nst-1(RNAi)*	100	0	>100
*nst-1(vr6); vrEx5*	95	5	>38
*nst-1(vr6); vrEx6*	0	0	>100
*nst-1(vr6); vrEx31*	0	0	>50

To determine a potential cause for the larval arrest, we asked if the *nst-1(vr6)* mutants exhibited a growth defect. We measured the length of *nst-1(vr6)* and wild type larvae from head to tail at multiple timepoints during larval development, beginning immediately after hatching as previously described [13]. The genotypes were indistinguishable in length for the first four hours of post-embryonic development, after which point *nst-1(vr6)* larvae failed to grow significantly (Figure 1C; n = 6). This growth defect precedes essentially all postembryonic cell divisions, suggesting that it is not solely due to defective larval cell divisions, but also a failure of existing cells to support growth.

To ascertain whether *nst-1(vr6)* mutants undergo any normal larval blast cell divisions, we looked at the division of intestinal cells. At hatching, wild-type animals have 20 intestinal cells, 14 of which divide at the L1 molt resulting in 34 intestinal nuclei. *nst-1(vr6)* mutants also have 20 intestinal cells at hatching, but these cells do not divide at the L1 molt as seen in controls (20 versus 31 cells at 16 hours post hatching; n≥9). We next examined the division of germ cells in *nst-1(vr6)* mutants during L1 development. In order to distinguish germ cells from somatic cells, we used a strain where P granules are marked by RFP. We found that the precursor germ cells Z_2_ and Z_3_ are present in PGL-1::RFP; *nst-1(vr6)* mutants similar to controls (n≥10). Eight hours post hatching, PGL-1::RFP; *nst-1(vr6)* animals had significantly less germ cells than control PGL-1::RFP animals (3.7 versus 7.1; p<6.34×10^−7^; n≥7). The reduction in germ cell divisions in PGL-1::RFP; *nst-1(vr6)* mutants was also evident at the L1 molt (5.5 versus 12 in PGL-1::RFP controls; p<1.5×10^−9^; n = 10). Therefore, the *nst-1* lesion causes larval arrest due to a defect that occurs very early during post-embryonic development, and which subsequently may impair later larval cell divisions in both the germ line and the soma.

### *nst-1* Acts in the Germ Line to Promote Germ Cell Proliferation

Because nucleostemin has a conserved function in modulating cell proliferation [14], we wanted to examine the effects of loss of *nst-1* in the germ line, which contains proliferating germ cells in both larvae and adults. We found that brood size was significantly decreased in *nst-1(vr6)/*mIn1 heterozygotes compared to +/mIn1 (188 versus 260, p<1.93×10^−5^) or +/+ animals (188 versus 291, p<2.11×10^−7^; n≥11), which might be indicative of impaired proliferation. However, the larval arrest phenotype of *nst-1(vr6)* homozygous mutants prior to extensive germ cell proliferation prevents direct analysis of this issue.

**Figure 2:**
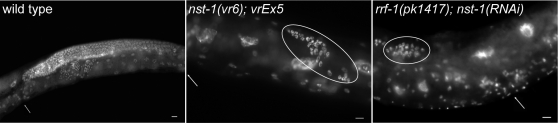
*nst-1* is required in the germ line for germ cell development. Wild type, *nst-1(vr6); vrEx5*, and *rrf-1(pk1417); nst-1(RNAi)* progeny adults were stained for DAPI to visualize nuclei. Germ cells in one gonad arm of *nst-1(vr6); vrEx5* and *rrf-1(pk1417); nst-1(RNAi)* animals are circled. Arrows indicate the vulva. Scale bars are 10 µm.

To overcome this limitation, we took two complementary approaches to rescue the larval arrest in the soma and selectively investigate the *nst-1* mutant phenotype in the germ line. In the first approach, we took advantage of the fact that high-copy, repetitive extrachromosomal transgenes are silenced in the germ line, but are still expressed in the soma [15]. We created an extrachromosomal transgenic line, *vrEx5*, that expresses wild-type *nst-1* under the control of its endogenous regulatory elements. We generated *nst-1(vr6); vrEx5* animals, and found that these animals did not arrest as larvae, but instead grew up to become sterile adults (Table 1). The germ lines of these soma-rescued mutants were severely underproliferated, with only a small percentage containing sperm (10.5%) and none containing oocytes at 20°C (n = 38; Figure 2).

In the second approach, we selectively removed *nst-1* function from the germ line in otherwise wild-type animals by performing *nst-1(RNAi)* in *rrf-1(pk1417)* mutants, which are RNAi-defective in the soma, but RNAi-competent in the germ line [16]. *rrf-1(pk1417); nst-1(RNAi)* animals also overcame the larval arrest and reached adulthood, but were sterile with severe defects in proliferation and gamete differentiation in the germ line, similar to the phenotype of *nst-1(vr6); vrEx5* animals (Figure 2, Table 1).

These experiments demonstrate that *nst-1* acts in the germ line to modulate germ cell development, but do not rule out the possibility that *nst-1* could still have an additional role in somatic tissue(s) that control germline stem cell proliferation.

### *nst-1(vr6)* Mutant Germ Lines Exhibit Decreased Proliferation

**Figure 3:**
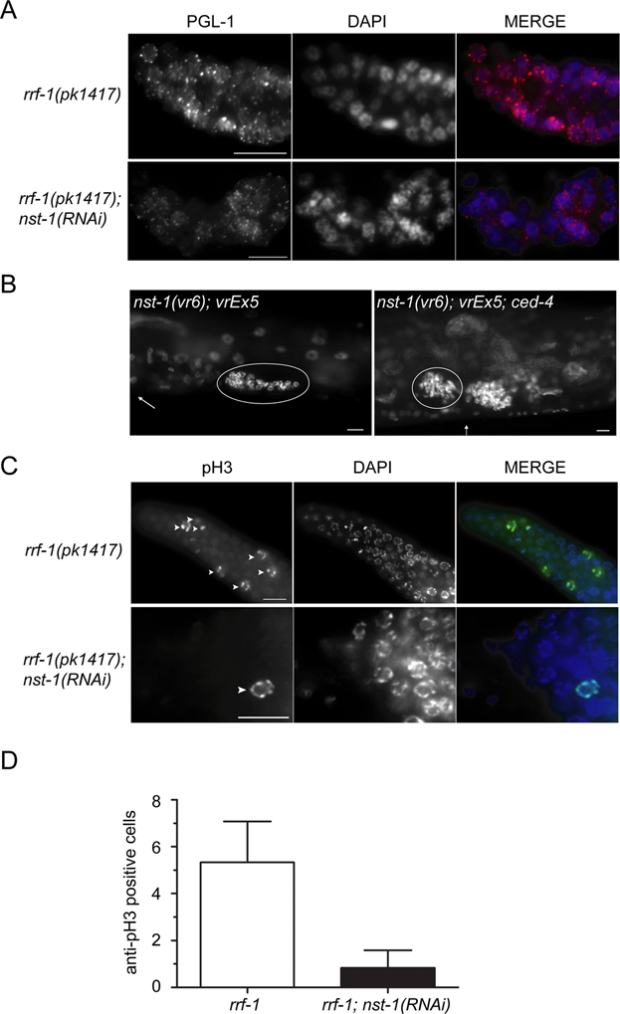
Germ cells lacking *nst-1* fail to proliferate. A. α-PGL-1 (red) and DAPI (blue) staining in dissected gonads of *rrf-1(pk1417)* controls and *rrf-1(pk1417); nst-1(RNAi)* progeny. Similar staining was seen in *nst-1(vr6); vrEx5* animals (not shown). B. *nst-1(vr6); vrEx5* and *nst-1(vr6); vrEx5; ced-4(n1162)dpy17(e164)* soma-rescued transgenic animals were stained with DAPI to visualize nuclei. Arrows indicate the vulva. Germ cells from one gonad arm are circled. C. α-pH3 (green) and DAPI (blue) staining in dissected gonads of *rrf-1(pk1417)* controls and *rrf-1(pk1417);nst-1(RNAi)* progeny. Arrowheads mark the α-pH3-positive cells in each genotype. D. Quantification of α-pH3-positive cells. Error bars indicate standard deviation (n = 6 per genotype). Scale bars are 10 µm.

We used both soma-rescued backgrounds, *nst-1(vr6); vrEx5* and *rrf-1(pk1417); nst-1(RNAi)*, for subsequent analyses of germ cell defects. First, we determined whether germ cells lacking *nst-1* maintained germ granules, a key characteristic of germ cells. Using an antibody to the core germ granule component PGL-1, we found that germ cells from both the soma-rescued transgenic line, *nst-1(vr6); vrEx5*, and from *rrf-1(pk1417); nst-1(RNAi)* progeny maintained perinuclear punctate staining of PGL-1 similar to *nst-1/*mIn1; *vrEx5* and *rrf-1(pk1417)* controls, respectively, suggesting that germ granules are likely to be intact (data not shown and Figure 3A).

We next wanted to determine why *nst-1* mutants have so few germ cells. Germ cells lacking *nst-1* either cannot proliferate, or they have a normal rate of proliferation coupled with excessive cell death by apoptosis or necrosis, possibly induced as a response to stresses inflicted by loss of *nst-1* function. We therefore placed the soma-rescued transgenic line, *nst-1(vr6); vrEx5*, in a *ced-4(n1162)* mutant background, which cannot undergo apoptosis [17]. The presence or absence of *ced-4* activity had no apparent effect on the germline phenotype of the *nst-1(vr6); vrEx5* animals (Figure 3B; n≥10). Consistent with our findings, mouse *NS* ^−/−^ embryos do not exhibit abnormal caspase-3 immunostaining [14]. Because prohibiting apoptosis did not increase germ cell number, the germline defects of the soma-rescued *nst-1(vr6); vrEx5* animals are likely due to decreased proliferation or some form of non-apoptotic cell death such as necrosis. However, the fairly normal morphology of *nst-1(vr6)* germ cells is not consistent with necrosis.

Therefore, to examine whether loss of *nst-1* results in decreased proliferation, we utilized an antibody against phosphorylated-histone H3 (pH3), which marks germ cells in late prophase and early mitotic M-phase [18]. We found a significant reduction in the number of α-pH3-positive cells in the *rrf-1(pk1417); nst-1(RNAi)* animals compared to *rrf-1(pk1417)* controls (Figure 3C, D; n = 6). In agreement with our results, mouse *NS* ^−/−^ embryos also have a reduced number of α-pH3-positive cells compared to controls [14]. We conclude that germ cells lacking *nst-1* do not have normal proliferation rates, and because they do not initiate apoptosis or appear to lose cell identity based on normal PGL-1 staining, we suggest that they undergo a cell cycle arrest.

### Germ Cells Lacking *nst-1* Are Able to Differentiate into Sperm

Germ cells in both the soma-rescued transgenic line and in the *rrf-1(pk1417); nst-1(RNAi)* progeny undergo very little differentiation into gametes, with only 10% of the soma-rescued *nst-1(vr6)* mutants containing sperm. The failure to differentiate into gametes could be an intrinsic failure of the germ cells to be able to progress through meiosis and differentiation. Another possibility is that the *nst-1(vr6)* mutant germ cells remain too close to the distal tip cell, which expresses the LAG-2 ligand that activates GLP-1 signaling in germ cells to prevent differentiation (Figure S2; n>9)[19].

To determine whether *nst-1* mutant germ cells were capable of differentiating into sperm if the GLP-1-mediated block to differentiation was removed, we utilized the temperature sensitive *glp-1* mutant, *glp-1(e2141)*. This mutant is fertile at 15°C, but when shifted to the restrictive temperature of 25°C after hatching, all germ cells prematurely enter meiosis and differentiate into sperm [20]. We placed the *glp-1(e2141)* mutation in the background of *nst-1(vr6); vrEx5* and analyzed germ cell differentiation. Germ cells lacking *nst-1* effectively differentiate into sperm when the block to meiosis is removed, compared to controls retaining one copy of *nst-1* in the *glp-1(e2141)* mutant background (4.9±3.9 versus 4.5±1.4 sperm per animal; p = 0.578; n>45). Thus, *nst-1(vr6)* mutant germs cells are capable of differentiation, but their failure to migrate a sufficient distance from the distal tip cell prevents them from doing so.

### Loss of *cep-1* Does Not Rescue the *nst-1(vr6)* Mutant Phenotype

Mammalian nucleostemin (NS) interacts with p53 in pull-down assays [9] and has been shown to modulate the G1/S transition of the cell cycle via the p53 pathway in culture [21]. However, loss of p53 was not sufficient to rescue the embryonic lethality of *NS^−/−^* mice [14], making the relevance of an interaction between NS and p53 in vivo less clear. To determine whether NST-1 might interact with p53, known as *cep-1* in *C. elegans*, we first tested whether loss of *cep-1* could rescue the larval arrest phenotype of *nst-1(vr6)* mutants. We found that *cep-1(gk138); nst-1(vr6)* mutants still exhibited a larval arrest phenotype (data not shown). We also tested whether *cep-1* played a role in the *nst-1* germline phenotype, and found that *cep-1(gk138); nst-1(vr6); vrEx5* mutants exhibited an under-proliferation phenotype in the germ line very similar to that of *nst-1(vr6); vrEx5* mutants alone (n≥38; data not shown). Thus, loss of *cep-1*/p53 does not have an obvious effect on the *nst-1(vr6)* mutant phenotype in either the soma or germ line. This lack of rescue is consistent with the inability of p53 loss to rescue the embryonic lethality of mouse NS [14].

### *nst-1(vr6)* Mutants Exhibit Defects in Ribosome Biogenesis

**Figure 4:**
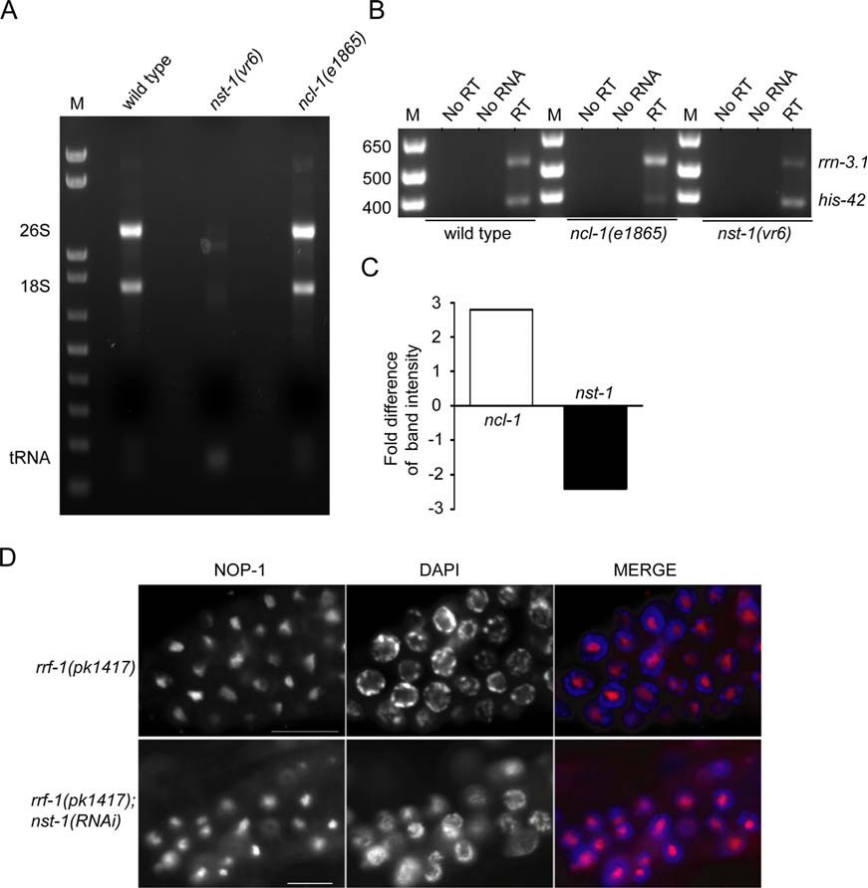
*nst-1(vr6)* mutants have aberrant rRNA levels. A. 1 µg of total RNA from wild type, *nst-1(vr6)*, and *ncl-1(e1865)* L1-staged animals was loaded on a 1% agarose gel and stained with ethidium bromide. M = marker. B. RT-PCR of 26S rRNA (*rrn-3.1*) in wild type, *nst-1(vr6,)* and *ncl-1(e1865)* L1 animals. Histone H2B (*his-42*) served as a loading control. M = marker. C. The fold difference of band intensity compared to wild type. D. α-NOP-1/fibrillarin (red) and DAPI (blue) staining in dissected control *rrf-1(pk1417)* and *rrf-1(pk1417); nst-1(RNAi)* germ lines. Similar staining was observed in *nst-1(vr6); vrEx5* animals (not shown). Scale bars are 10 µm.

Loss of NST-1 in the soma or germ line causes cell growth and proliferation defects. These phenotypes may be the result of a role for NST-1 in modulating ribosome biogenesis, based on studies of NUG1, the nucleostemin ortholog in yeast. NUG1 exports pre-60S ribosomal subunits out of the nucleolus and when mutated, cell growth is impaired [12]. To ask whether *C. elegans nst-1* has a role in ribosome biogenesis, we examined rRNA abundance in wild type, *nst-1(vr6)*, and *ncl-1(e1865)* L1 animals. *ncl-1(e1865)* mutants exhibit elevated levels of rRNAs [22] and served as a control. We examined young L1 animals prior to the onset of the larval arrest phenotype to avoid effects on ribosome biogenesis that might be downstream consequences of the growth defect. Using gel electrophoresis of equal amounts of total RNA, we consistently saw decreased 18S and 26S rRNA levels, with correspondingly higher levels of tRNA, in *nst-1(vr6)* mutants compared to wild type (Figure 4A). rRNA levels in *ncl-1(e1865)* mutants appeared higher than in wild type, consistent with published reports [22].

To independently confirm the decreased rRNA levels seen in the *nst-1(vr6)* mutant, we performed RT-PCR amplification of *rrn-3.1*, which encodes a 26S rRNA. Consistent with the total RNA analysis, *rrn-3.1* levels are significantly lower in *nst-1(vr6)* mutants compared to wild-type animals (2.7-fold, ±0.4, from two independent RT-PCR experiments) (Figure 4B, C). The decreased rRNA levels seen in the *nst-1(vr6)* mutant suggests that ribosome biogenesis is not occurring at normal levels, a defect that possibly underlies the larval arrest phenotype.

The decreased rRNA levels in *nst-1(vr6)* mutants led us to examine whether nucleoli were aberrant. We found no apparent difference in size (p<0.22; n≥18) or morphology of intestinal nucleoli between mutant and wild-type newly-hatched larvae. Additionally, to determine if germ cells lacking *nst-1* have aberrant nucleoli, we stained the dissected gonads of *rrf-1(pk1417); nst-1(RNAi)* progeny with NOP-1/fibrillarin, a specific nucleolar marker. We did not detect any gross differences between the *rrf-1(pk1417); nst-1(RNAi)* and control *rrf-1(pk1417)* progeny (Figure 4D; n≥7), or in the soma-rescued *nst-1(vr6); vrEx5* transgenic line (data not shown). Our result is consistent with the finding that mouse *NS^−/−^* mutant embryos have normal nucleolar morphology, based on fibrillarin staining [14].

To determine whether increasing the levels of pre-rRNA might rescue the larval arrest phenotype of *nst-1(vr6)* mutants, we generated an *nst-1(vr6); ncl-1(e1865)* double mutant. *ncl-1* acts as a repressor of rRNA transcription and the *ncl-1(e1865)* mutant contains 1.6-fold more rRNA than wild type, resulting in larger nucleoli [22]. The *nst-1(vr6); ncl-1(e1865)* double mutant still exhibited larval arrest and growth defects comparable to *nst-1(vr6)* mutants (Figure S3; n≥5). We also found that the nucleoli of *nst-1(vr6); ncl-1(e1865)* mutants were not statistically different in size from *nst-1(vr6)* mutants alone (p<0.114; n≥12). Thus, increasing endogenous pre-rRNA levels does not rescue the defects of loss of *nst-1*. These results suggest that *nst-1* acts in ribosome biogenesis downstream or independently of *ncl-1*.

### NST-1 Is a Nucleolar Protein

**Figure 5:**
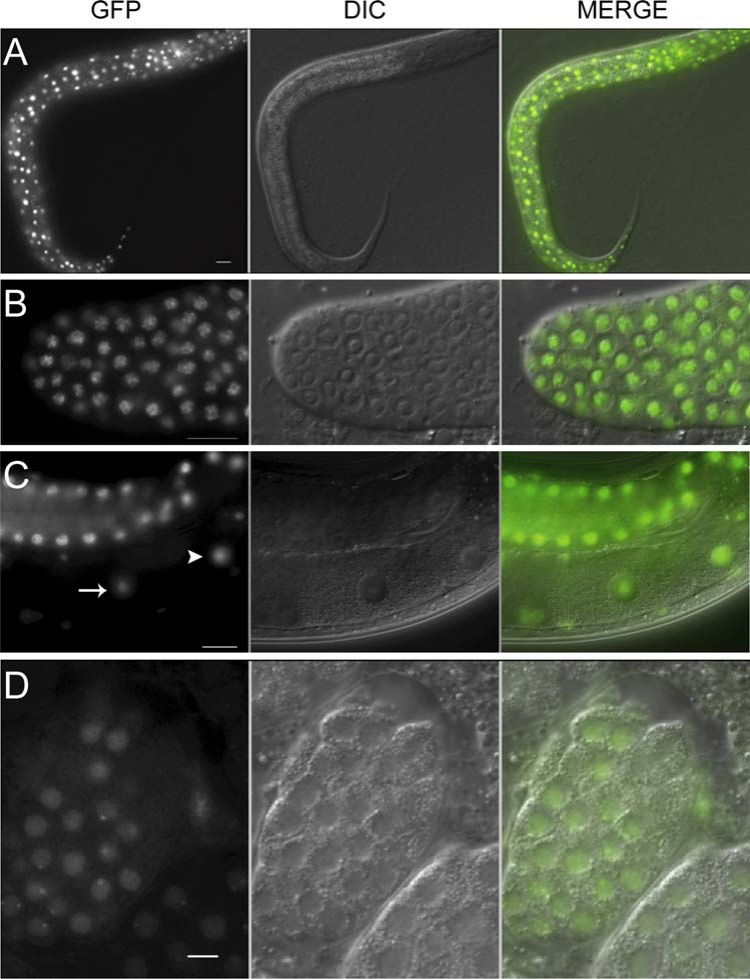
NST-1 is broadly expressed and localized to the nucleolus. A. DIC and GFP image of *unc-119(ed3) III; vrEx6 [nst-1::GFP, unc-119 (+)]* L2 larva. B. DIC and GFP image of dissected gonad distal tip showing NST-1 expression in the mitotic region. C. Whole animal DIC and GFP image illustrating the decreased expression of NST-1 in the most proximal oocyte indicated by arrow. The distal oocyte is indicated by the arrowhead. D. Expression in a ≥20-cell embryo. Scale bars are 10 µm.

To assess the subcellular localization of NST-1 in vivo, we generated two independent transgenic lines expressing the NST-1 protein fused to GFP (NST-1::GFP) under the control of endogenous *nst-1* regulatory elements. The use of microparticle bombardment to generate low-copy transgenic lines permitted expression of this transgene in the germ line. Both transgenes can rescue *nst-1(vr6)* mutants to fertile adulthood with brood sizes similar to the *nst-1* heterozygote, indicating that both somatic and germ line defects were rescued. Both strains showed similar NST-1 expression and localization. During post-embryonic development, NST-1::GFP was ubiquitously expressed in the soma and germ line from L1 larvae to the adult stage (Figure 5A). It is concentrated in the nucleolus and diffusely present in the nucleoplasm, similar to reports of mammalian nucleostemin localization (Figure 5B) [9]. It was uniformly expressed in all cells of the adult oogenic germ line until the proximal oocyte, where expression decreased (Figure 5C). Expression was not detected in the early embryo until the ≃18-cell stage, when it again became detectable in the very small nucleolus and diffusely present in the nucleoplasm (Figure 5D). The lack of NST-1::GFP expression in the early embryo is consistent with the fact that rRNA processing and assembly of newly produced ribosomes are not occurring at this time [23], although rRNA transcription is detectable [24].

### NST-1 Does Not Exclusively Reside within the rRNA Processing Center

**Figure 6:**
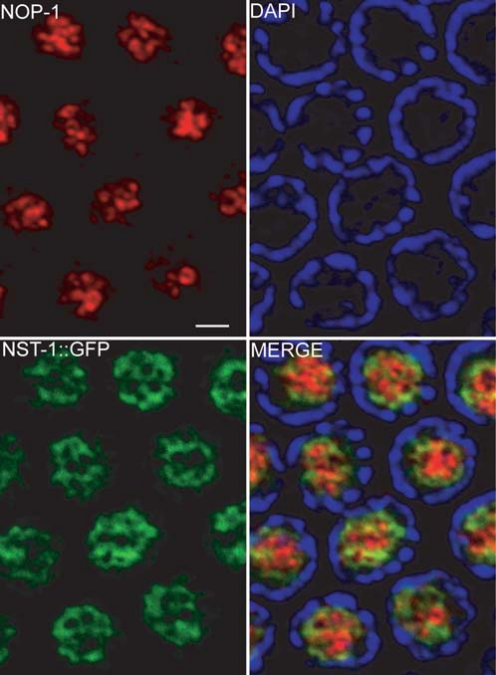
NST-1 and NOP-1 primarily occupy different domains within the nucleolus. Dissected gonads of *unc-119(ed3) III; vrEx6 [nst-1::GFP, unc-119 (+)]* co-stained with α-NOP-1 (red), α-GFP (green), and DAPI (blue). Scale bar is 2 µm.

In order to determine more precisely the localization of NST-1 within the nucleolus, we examined the co-localization of NST-1::GFP with NOP-1/fibrillarin in the germ line. NOP-1 is a specific marker for the dense fibrillar component and is directly involved in rRNA processing [25]. We observed obvious regions within the nucleolus where NOP-1 was highly expressed and NST-1::GFP was completely absent (Figure 6). Overlap between NOP-1 and NST-1::GFP occurred primarily in areas where NOP-1 was expressed at relatively low levels, suggesting that NST-1 does not reside in regions of robust rRNA processing. This minimal co-localization is consistent with a failure of mammalian nucleostemin to significantly co-localize with fibrillarin [11]. Although we found that loss of *nst-1* results in lower rRNA levels, the absence of NST-1 in regions of the nucleolus where rRNA processing occurs suggests that NST-1 is not likely to play a direct role in rRNA processing and that the effect on rRNA levels may be secondary.

### The GTPase and Intermediate Domains Are Required for NST-1 Function in the Germ Line

NST-1 and mammalian nucleostemin are highly homologous in the predicted functional domains, especially the basic domain and the two GTPase domains, G1 GXXXXGK[S/T] and G4 KXDL (Figure 1B). In order to determine the importance of the functional domains of NST-1, we made point mutations and/or deletions within the rescuing NST-1::GFP construct and assessed the effects on subcellular localization and the ability to rescue the *nst-1(vr6)* mutant.

**Figure 7:**
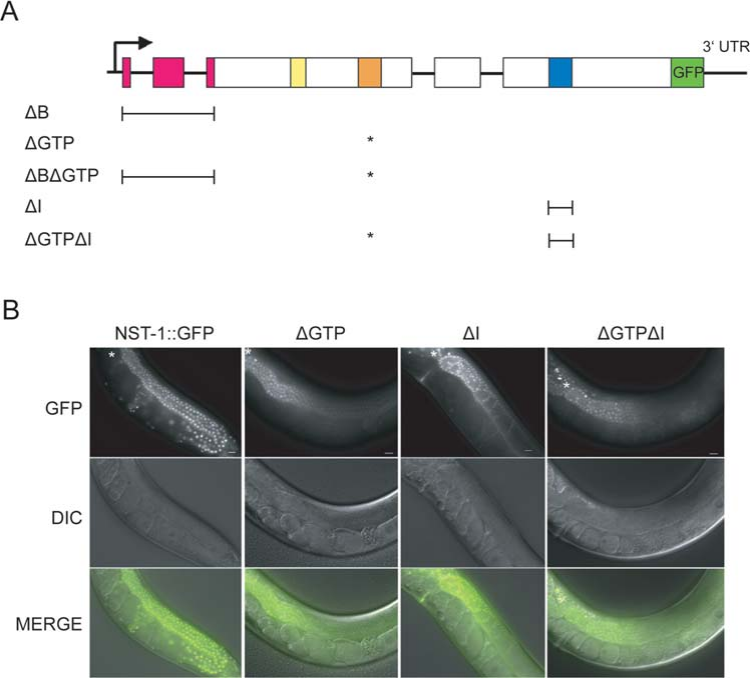
Mutations in NST-1::GFP alter the spatial localization of NST-1 in the germ line. A. Schematic of the mutations in NST-1::GFP. The asterisk marks the location of the G1 GTPase point mutation. The brackets indicate the region deleted in NST-1::GFP. B. Whole animal Nomarski images comparing the spatial expression patterns of NST-1::GFP lines in the germ line. NST-1::GFP is ubiquitously expressed in the distal and proximal germ line, while the NST-1(ΔGTP)::GFP, NST-1(ΔI)::GFP, and NST-1(ΔGTPΔI)::GFP transgenic lines have higher expression in the distal end compared to the proximal end. Asterisk marks the distal tip. Scale bars are 10 µm.

The GTP-binding capacity of mammalian nucleostemin is dependent upon the G1 motif [10], which is identical between mammals and *C. elegans*. A single amino acid substitution, G256V, in mammalian nucleostemin decreases its GTP-binding activity in vitro, and causes aberrant localization of nucleostemin and formation of nucleolar aggregates [10]. We inserted the equivalent amino acid substitution in the wild-type rescuing NST-1::GFP construct (called ΔGTP) and obtained three independent lines (Figure 7A). We did not observe any changes in subcellular localization: NST-1 (ΔGTP)::GFP still localized normally to the nucleolus and nucleoplasm, and did not form obvious aggregates. However, the spatial distribution of NST-1(ΔGTP)::GFP was no longer ubiquitous in the germ line but exhibited higher expression in the distal end compared to the proximal end, with an abrupt downregulation at the point of entry into meiosis (Figure 7B, Table 2). Additionally, expression in the soma became limited to a subset of tissues such as seam cells and body wall muscle (Table 2). We speculate that the difference in spatial expression of NST-1 may be due to protein de-stabilization, with turnover occurring more rapidly in the proximal region of the germ line.

**Table 2:** Summary of NST-1::GFP mutation transgenic lines.

	% Lines w/expression*	Somatic expression	Germline expression	Subcellular localization	Rescues lvaˆ	Rescues sterilityˆ
NST-1::GFP	71(14)	ubiquitous	ubiquitous	nucleolar**	100%	100%
ΔB	0(11)	NA	NA	NA	NA	NA
ΔGTP	88(24)	broad	higher in distal end	nucleolar**	61%ˆˆ	0%ˆˆ
ΔBΔGTP	0(5)	NA	NA	NA	NA	NA
ΔI	76(34)	moderately restricted	higher in distal end	nucleolar**	22%	0%
ΔGTPΔI	30(10)	moderately restricted	higher in distal end	nucleolar**	0%	0%

NA = not applicable.

Lva = larval arrest.

*: Number of lines in parentheses.

**: Expression is primarily nucleolar and diffuse in the nucleoplasm.

ˆ Based on two independent extrachromosomal lines crossed to *nst-1(vr6); unc-119(ed3)*.

ˆˆ Based on three independent extrachromosomal lines crossed to *nst-1(vr6); unc-119(ed3)*.

When we crossed the ΔGTP transgene into *nst-1(vr6)* mutants, we found that all three lines rescued the larval arrest phenotype of *nst-1(vr6)* mutants; however the animals were sterile with severely underproliferated germ lines (Table 2). The ability of the ΔGTP construct to rescue the somatic growth defects but not the germ cell proliferation defects, despite expression in the distal region of the germ line, argues that this domain is not necessary for NST-1 function in the soma but is necessary in the germ line.

In mammals, the N-terminal basic region is important for nucleolar localization of NS; when the region was removed, the protein became more diffuse [9]. We made a similar deletion in NST-1::GFP (called ΔB) (Figure 7A). We obtained eleven independent lines and never observed NST-1(ΔB)::GFP expression (Table 2). We also generated a construct with both the ΔGTP and ΔB deletions, and could not detect any NST-1 expression in five lines (Figure 7A, Table 2). These data suggest that lack of the basic region renders the protein unstable.

When the intermediate domain was removed from NST-1:GFP (called ΔI), we again observed that subcellular localization was unaffected, and the spatial expression of NST-1 was higher in the distal end compared to the proximal end, as in NST-1(ΔGTP)::GFP (Figure 7A, B). The number of cells that expressed NST-1(ΔI)::GFP in the soma was less than that seen in NST-1(ΔGTP)::GFP (Table 2). Two independent lines of NST-1(ΔI)::GFP were able to rescue the larval arrest phenotype in 22% of *nst-1(vr6)* mutants, although this rescue was not nearly as robust compared to the ΔGTP lines (61%; Table 2). The few soma-rescued animals were sterile with very few germ cells, indicating that the intermediate domain is required for germ cell proliferation. Its ability to rescue only a fraction of the *nst-1(vr6)* mutants may be because NST-1(ΔI)::GFP had limited expression in somatic cells in larvae, and thus was not expressed in the correct cells to permit rescue.

For mammalian NS, simultaneously removing the intermediate domain and inserting the G256V point mutation caused the protein to reside exclusively within the nucleolus [10]. When similar dual mutations were made in NST-1::GFP, the subcellular localization of NST-1(ΔGTPΔI)::GFP remained normal, with primarily nucleolar and faint nucleoplasmic localization (Figure 7A, Table 2). Again, the spatial distribution of NST-1(ΔGTPΔI)::GFP in the germ line was higher in the distal end compared to the proximal end (Figure 7B), and showed moderately restricted expression in the soma, similar to the ΔI lines. However, NST-1(ΔGTPΔI)::GFP never rescued the larval arrest phenotype of *nst-1(vr6)* mutants (Table 2). We suggest that the combination of the ΔGTP and the ΔI mutations renders NST-1 non-functional in both the soma and the germ line.

### Mammalian NS Does Not Rescue *nst-1(vr6)* Mutants

In order to determine if mammalian NS could rescue *nst-1(vr6)* mutants, we generated two independent transgenic lines expressing mNS::GFP under the control of *C. elegans* endogenous regulatory elements using microparticle bombardment. Both transgenes are extrachromosomal and express detectable protein that is localized to the nucleolus, similar to NST-1::GFP, and the transgenic animals are healthy. However, neither mNS:GFP transgenic line was able to rescue the larval arrest of *nst-1(vr6)* mutants. This observation suggests that functional differences exist between mouse and *C. elegans* nucleostemin.

## Discussion

Our analysis of *nst-1* function in the soma and in the germline stem cells of *C. elegans* has revealed a role for *nst-1* in modulating cell growth and proliferation. We found that loss of *nst-1* in the soma leads to larval arrest and cell growth defects, while a lack of *nst-1* in the germ line causes germ cells to undergo a cell cycle arrest. Our work provides several lines of evidence suggesting that *nst-1* controls proliferation and cell growth by regulating ribosome biogenesis: (1) NST-1 is required for larval development but appears dispensable for embryogenesis, similar to other factors important for ribosome biogenesis that are not ribosome subunits themselves, such as RBD-1 and FIB-1 [23], (2) ribosomal RNA production is significantly reduced prior to any other detectable phenotype in *nst-1(vr6)* mutants, indicating that it is a component of the initial defect and not a secondary consequence, (3) NST-1 is clearly related to yeast NUG1, which has a demonstrated role in ribosome subunit export [12] and (4) NST-1 is expressed in the nucleolus where ribosome biogenesis occurs.

### How Does *nst-1* Affect rRNA Levels?

We have shown by two independent methods that *nst-1(vr6)* mutants have significantly reduced rRNA levels. The decreased rRNA levels seen in the *nst-1(vr6)* mutant are likely not due to a role for NST-1 processing, because NST-1 is absent from regions of robust rRNA processing within the nucleolus. In yeast, the related protein NUG1 has been shown to export RPL25.2, a pre-60S subunit, out of the nucleolus [12]. We hypothesize that NST-1 also shuttles partially assembled ribosomes in and out of the nucleolus. In the absence of functional NST-1, the ribosomal subunits would remain within the nucleolus, and ultimately affect global rRNA levels due to a negative feedback mechanism that results in decreased ribosome biogenesis. We attempted to test this possibility by determining whether a GFP-tagged ribosome subunit, RPL-25.2, was restricted to the nucleolus in *nst-1(vr6)* mutants. However, even low expression levels of RPL-25.2::GFP made the animals too sick to analyze.

### How Conserved Are the Functional Domains of Nucleostemin?

Mutations in the highly conserved functional domains of NST-1 did not result in changes in subcellular localization seen in mammalian nucleostemin studies. We did however observe alterations in the spatial expression in the germ line for the ΔGTP, ΔI, and ΔGTPΔI mutations in NST-1::GFP. We suggest that the alteration in spatial expression of NST-1 may be due to moderate de-stabilization of the mutant proteins. The NST-1(ΔGTP)::GFP and NST-1(ΔI)::GFP transgenic lines are at least partially able to rescue the somatic defects of *nst-1(vr6)* mutants, arguing that neither domain is absolutely essential in the soma. The inability of the NST-1(ΔGTPΔI )::GFP transgenic line to rescue the somatic phenotype of *nst-1(vr6)* mutants may be due to incomplete expression, although we believe this is unlikely because it is expressed as broadly as the ΔGTP lines that do rescue this phenotype. Rather, we think it more likely that losing both the G1 GTPase domain and the intermediate domain in combination is more deleterious than loss of either alone.

Interestingly, the NST-1(ΔGTP)::GFP and NST-1(ΔI)::GFP transgenic lines are not able to rescue the germline defects of *nst-1(vr6)* mutants, despite being expressed in the germ line. This observation argues that both domains are necessary for NST-1 function in the germ line, and suggests that the functional domains of NST-1 may have different roles in the soma and germ line. Another possibility is that NST-1 needs to be ubiquitously expressed in the germ line to maintain proper germ cell development.

### Does Nucleostemin Have a Conserved Role in Modulating Proliferation by Regulating Ribosome Biogenesis?

To date, it has been shown that *S. cerevisae*, *S. pombe*, and, in this work, *C. elegans* nucleostemin modulates cell growth and proliferation by regulating ribosome biogenesis. The *S. pombe* NUG1 homologue Grn1 is involved in 60S biogenesis, and Grn1 mutants have a severe growth defect with a significant reduction in mature rRNA species similar to the *C. elegans nst-1(vr6)* mutant phenotype [26]. It has not been elucidated whether mammalian nucleostemin regulates some aspect of ribosome biogenesis, or whether it has diverged to have an additional function that is specific to stem cells or other rapidly proliferating cells. The expression of mammalian nucleostemin is apparently restricted to proliferating cells, but our studies show that in *C. elegans*, NST-1 is expressed in terminally differentiated cells as well as in proliferating cells. Moreover, the effect of mutation of individual domains on localization and function is different between *C. elegans* and mammals. This difference in regulation suggests that perhaps the role of nucleostemin is more limited or specialized in mammals. Consistent with this possibility, we were unable to rescue the *nst-1(vr6)* mutant phenotype using mouse nucleostemin cDNA, despite having a similar expression pattern and levels to NST-1::GFP, which suggests that there may indeed be functional differences between mouse and *C. elegans* nucleostemin.

Due to the fundamental requirement for translation in living cells, it has been difficult to determine exactly how ribosome biogenesis is specifically connected to cell growth and proliferation. Nucleostemin is potentially a key link between these two processes, as it could regulate the rate of translation to cell growth by modulating the rate of 60S subunit formation in response to cell-extrinsic cues. Further studies directed toward identifying the cargo of nucleostemin and the cell cycle-specific mechanisms controlling its ability to shuttle in and out of the nucleolus will likely shed light on this key question.

## Materials and Methods

### Strains and Maintenance

Nematode strain maintenance was as described [27]. *C. elegans* strain N2 was used as the wild type strain in addition to the following variants: LGI, *cep-1(gk138)*, *gld-1(q485)*; LGII, *nst-1(vr6)*; LG III, *glp-1(e2141)*, *ced-4(n1162)*, *unc-119(ed3)*, *ncl-1(e1865)*, *unc-36(e251)*, *rrf-1(pk1417)*; LGV, *qIs19(lag-2*::GFP). The *unc-119(ed3); nym-2::PGL-1::mRFP* strain was a gift of James R. Priess. All experiments were conducted at 20°C unless otherwise indicated.

### Deletion Mutant Identification

To isolate mutations in K01C8.9 (*nst-1*), a library of mutagenized worms was screened for deletion alleles by PCR. The deletion library was constructed and screened as described [28]. Deletion breakpoints in *nst-1(vr6)* are GTCGCAAAAGCATCGAAACA / TTTTGAACAACACTGAGACC. After deletion mutations were identified, frozen worms from corresponding wells were recovered and homozygous mutants were isolated. Prior to phenotypic and genetic analysis, *vr6* was backcrossed to wild type six times to remove background mutations. Due to the larval arrest phenotype, *vr6* was balanced with the mIn1*[mIs14 dpy-10(e128)]* chromosome, which marks the pharynx with GFP.

### RNAi

RNAi was performed by injection as described [29]. *nst-1* dsRNA was prepared by in vitro transcription of PCR products amplified by primers with T7 sites. Primer sequences to amplify a 998 base pair region of *nst-1* were: 5′-taatacgactcactatagggCAATTCCCGACAATTGCTTT-3′ and 5′- taatacgactcactatagggGGCCCTTTCACTTTTCTTCC-3′. The concentration of injected dsRNA was 600–1000 ng/µl. After injection, hermaphrodites were allowed to lay eggs for 24 hours. Only progeny produced after this period were analyzed for larval arrest or sterility by comparing to controls and assessed by DAPI staining.

### Total RNA and RT-PCR

Total RNA from wild type, *nst-1(vr6)*, and *ncl-1(e1865)* L1-staged animals was extracted using Trizol (Invitrogen, Carlsbad, CA). Equal amounts of total RNA (1 µg) were electrophoresed on a 1% agarose gel and then stained with ethidium bromide. For RT-PCR, total RNA from wild type, *nst-1(vr6)*, and *ncl-1(e1865)* L1-staged animals was extracted using Trizol (Invitrogen, Carlsbad, CA) and samples were DNase treated with DNA-free (Ambion, Austin, TX). 100–150 ng of total RNA from each genotype was reverse transcribed using the Omniscript RT kit (Qiagen, Valencia, CA) and gene-specific PCR was performed using primers for *rrn-3.1*. *his-42* was used as a loading control. Samples were electrophoresed on a 2% agarose gel and then stained with ethidium bromide. Band intensities were measured using the spotdenso tool on AlphaEaseFC software (Alpha Innotech, San Leonardo, CA).

### Somatic Rescue Transgenic Construct

A genomic fragment containing the entire *nst-1* gene and its regulatory sequences (−497 to +1169 relative to the translational start site), a transformation marker P*_myo-3_*::GFP, which marks body wall muscle, and an empty vector pGEM5Z were co-injected into wild-type animals. Extrachromosomal lines were generated and crossed to the balanced strain *nst-1(vr6)/*mIn1. Animals that are GFP negative in the pharynx (*nst-1* homozygotes) and GFP positive in the body wall muscles (transgene positive) were assessed for larval arrest rescue and germline effects.

### Immunofluorescence

Gonads were dissected from animals 36–48 hours post L4 and fixed as described with the following antibodies and dilutions: affinity purified rabbit anti-PGL-1 (1∶30,000) (gift from S. Strome) [30], rabbit polyclonal anti-GFP (1∶200) (BD Biosciences, San Jose, CA), mouse anti-NOP-1/fibrillarin (1∶400) (EnCor Biotechnology Inc., Alachua, FL) [24], rabbit polyclonal anti-histone H3 phospho-S10 (1∶200) (Upstate, Billerica, MA) [31]. Samples were incubated at room temperature for 2–3 hours with a fluorescent secondary antibody (1∶500, Molecular Probes, Carlsbad, CA). Slides were mounted with anti-fade solution and viewed using a Zeiss Axioplan 2 imaging epifluorescence microscope.

### GFP Fusion Proteins

The same *nst-1* 5′ regulatory region and coding region as the somatic rescue transgenic construct (−497 to +1169 relative to the translational start site), the GFP coding region, and a larger *nst-1* 3′ regulatory region (+404 from the termination codon) were PCR amplified and stitched together as previously described [32]. The resulting PCR fragment was cloned into pCR2.1 TOPO (Invitrogen, Carlsbad, CA) and the *unc-119* rescuing genomic fragment with engineered NotI sites was ligated into the construct after NotI digestion. The resulting plasmid was transformed into *unc-119(ed3)* animals by microparticle bombardment as described [33] and extrachromosomal lines bearing a large percentage of non-Unc animals were examined for GFP expression. Deletions within the NST-1::GFP construct were made using PCR, and point mutations were inserted using the Gene Tailor Site-Directed mutagenesis system (Invitrogen, Carlsbad, CA). All constructs were sequenced prior to bombardment.

### Mammalian Rescue Construct

Full-length mouse NS cDNA was amplified from RNA extracted from mouse liver. The *C. elegans nst-1* regulatory elements and GFP were PCR amplified and stitched together with NS mouse cDNA as previously described [32]. The resulting PCR fragment was cloned into a plasmid containing the *unc-119* rescuing genomic fragment. The resulting plasmid was transformed into *unc-119(ed3)* animals by microparticle bombardment as described [33] and lines bearing a large percentage of non-Unc animals were examined for GFP expression.

## Supporting Information

Figure S1 RT-PCR analysis of *nst-1(vr6)* mutants detects reduced levels of truncated transcript. A. RT-PCR using gene specific primers 5′ of the *vr6* lesion in wild type and *nst-1(vr6)* L1-staged animals. Levels of the N-terminal transcript in *nst-1(vr6)* mutants were four-fold reduced compared to wild type. Hexokinase (*hexo*) served as a loading control. The asterisk marks genomic contamination. Lane 1 is the marker. Lanes 2 and 6 are empty. Lanes 3 and 7 are No RT controls. Lanes 4 and 8 are No RNA controls. Lanes 5 and 9 are the reverse transcribed experimental lanes. B. RT-PCR using gene specific primers 3′ of the *vr6* lesion (*nst-1* C-ter) and within the *vr6* lesion and outside of the vr6 lesion (*nst-1* deletion) in wild type and *nst-1(vr6)* L1-staged animals. Transcripts are not detected in *nst-1(vr6)* mutants using either of the primer sets. Hexokinase (*hexo*) served as a loading control. Lanes 1, 8, and 15 are markers. Lanes 2, 5, 9, and 12 are No RT controls. Lanes 3, 6, 10, and 13 are No RNA controls. Lanes 4, 7, 11, and 14 are the reverse transcribed experimental lanes.
(2.09 MB TIF)

Figure S2 *nst-1(vr6)* mutant germ cells are in close proximity to the distal tip cell. Nomarski images of wild type and *nst-1(vr6); vrEx5* animals with LAG-2:GFP expression. Note that *nst-1(vr6); vrEx5* animals carry the P_myo-3_::GFP transformation marker which marks body wall nuclei. The distal tip cell is marked by an asterisk. One gonad arm of *nst-1(vr6); vrEx5* is circled. The vulva is located in the bottom left of wild type and bottom right of *nst-1(vr6); vrEx5* animals. Scale bars are 10 µm.
(4.15 MB TIF)

Figure S3 The *nst-1(vr6)* larval arrest phenotype is not rescued by loss of *ncl-1. nst-1(vr6), nst-1(vr6)/*mIn1, *ncl-1(e1865)*, and *nst-1(vr6);ncl-1(e1865)* L1-staged animals were compared by measuring them head to tail. Error bars, standard deviation (n≥5).
(0.95 MB TIF)
