# Supplementary figures and images for: C. elegans Nucleostemin Is Required for Larval Growth and Germline Stem Cell Division
Source: PLoS Genet. 2008 Aug 22;4(8):e1000181. doi: 10.1371/journal.pgen.1000181 (PMC2515194; doi:10.1371/journal.pgen.1000181)

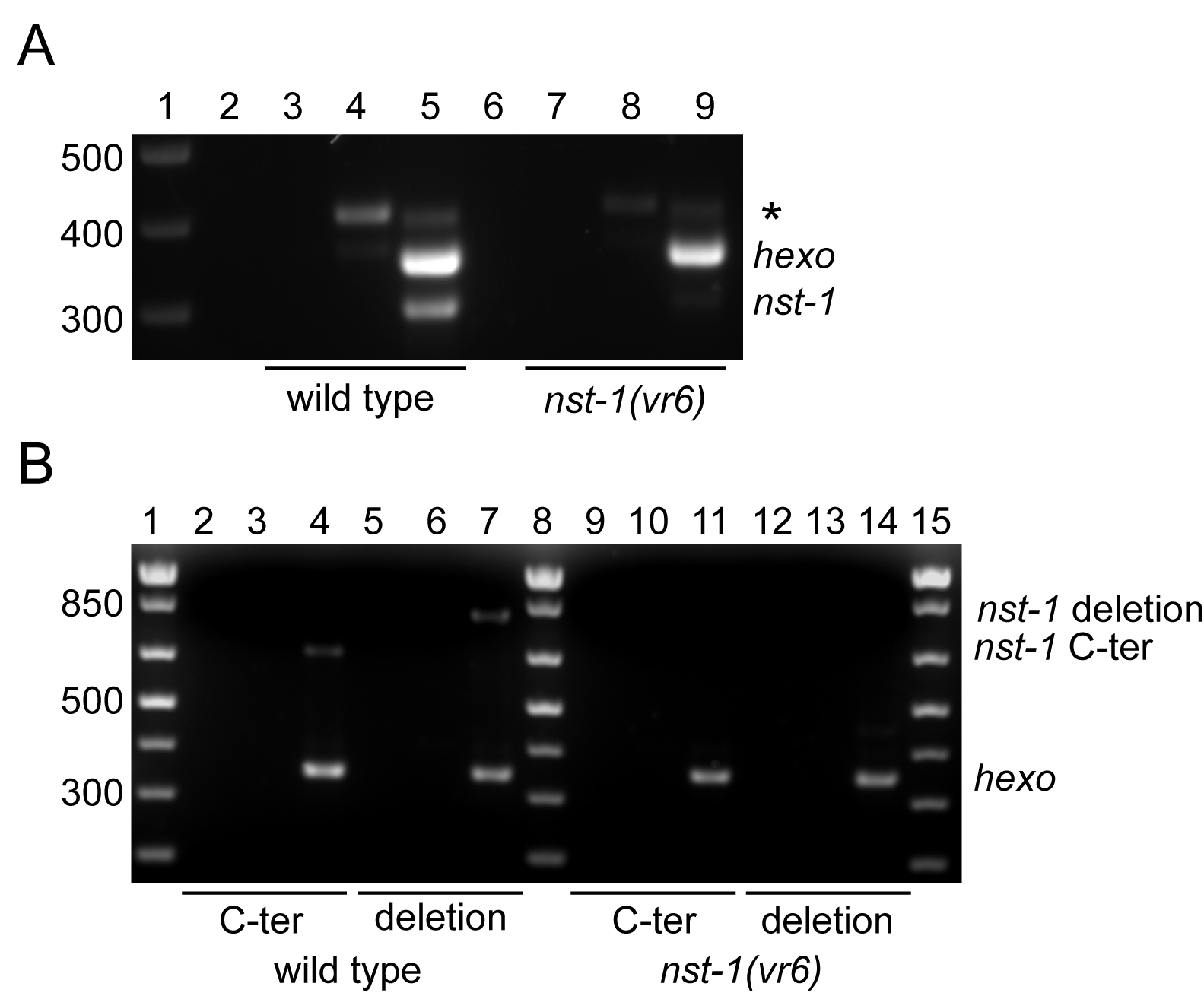

Supplement: Figure S1 — RT-PCR analysis of nst-1(vr6) mutants detects reduced levels of truncated transcript. A. RT-PCR using gene specific primers 5′ of the vr6 lesion in wild type and nst-1(vr6) L1-staged animals. Levels of the N-terminal transcript in nst-1(vr6) mutants were four-fold reduced compared to wild type. Hexokinase (hexo) served as a loading control. The asterisk marks genomic contamination. Lane 1 is the marker. Lanes 2 and 6 are empty. Lanes 3 and 7 are No RT controls. Lanes 4 and 8 are No RNA controls. Lanes 5 and 9 are the reverse transcribed experimental lanes. B. RT-PCR using gene specific primers 3′ of the vr6 lesion (nst-1 C-ter) and within the vr6 lesion and outside of the vr6 lesion (nst-1 deletion) in wild type and nst-1(vr6) L1-staged animals. Transcripts are not detected in nst-1(vr6) mutants using either of the primer sets. Hexokinase (hexo) served as a loading control. Lanes 1, 8, and 15 are markers. Lanes 2, 5, 9, and 12 are No RT controls. Lanes 3, 6, 10, and 13 are No RNA controls. Lanes 4, 7, 11, and 14 are the reverse transcribed experimental lanes. (2.09 MB TIF) [file pgen.1000181.s001.tif]

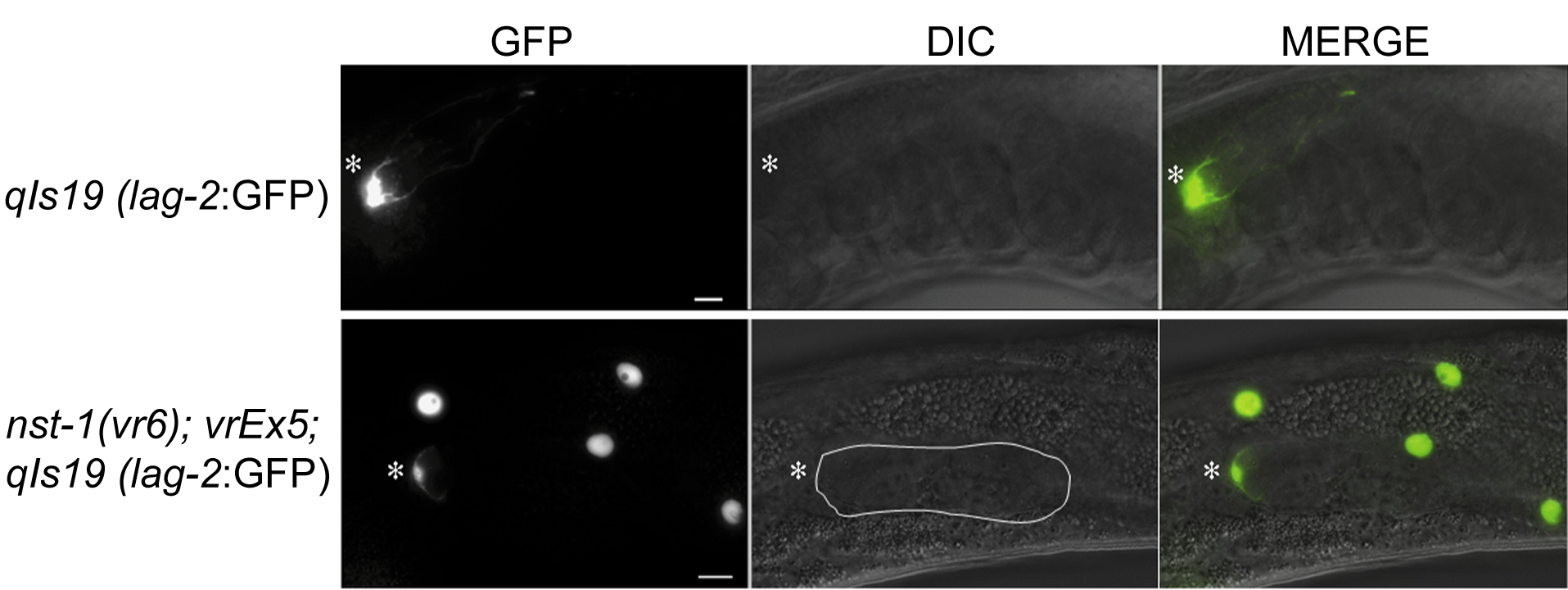

Supplement: Figure S2 — nst-1(vr6) mutant germ cells are in close proximity to the distal tip cell. Nomarski images of wild type and nst-1(vr6); vrEx5 animals with LAG-2:GFP expression. Note that nst-1(vr6); vrEx5 animals carry the Pmyo-3::GFP transformation marker which marks body wall nuclei. The distal tip cell is marked by an asterisk. One gonad arm of nst-1(vr6); vrEx5 is circled. The vulva is located in the bottom left of wild type and bottom right of nst-1(vr6); vrEx5 animals. Scale bars are 10 µm. (4.15 MB TIF) [file pgen.1000181.s002.tif]

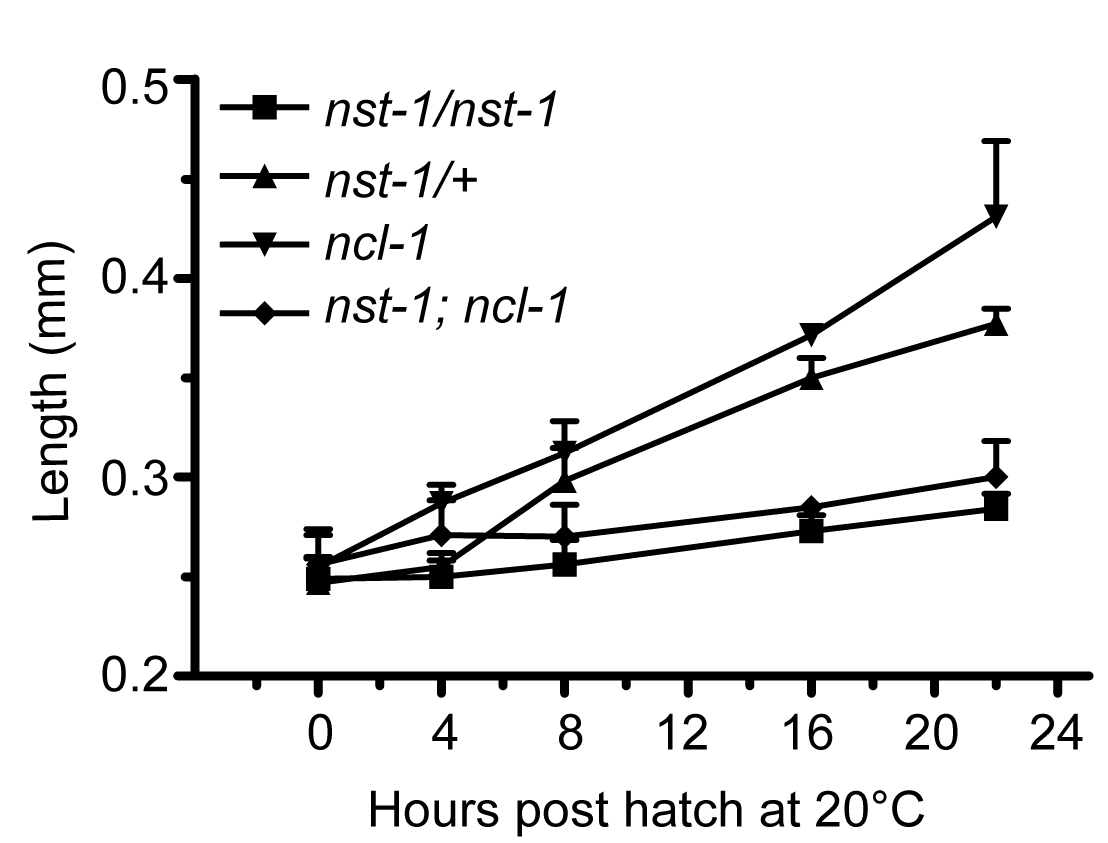

Supplement: Figure S3 — The nst-1(vr6) larval arrest phenotype is not rescued by loss of ncl-1. nst-1(vr6), nst-1(vr6)/mIn1, ncl-1(e1865), and nst-1(vr6);ncl-1(e1865) L1-staged animals were compared by measuring them head to tail. Error bars, standard deviation (n≥5). (0.95 MB TIF) [file pgen.1000181.s003.tif]
